# VEGFA promotes the occurrence of PLA2R-associated idiopathic membranous nephropathy by angiogenesis via the PI3K/AKT signalling pathway

**DOI:** 10.1186/s12882-022-02936-y

**Published:** 2022-09-16

**Authors:** Ben Ke, Jinjing Huang, Zhibing Duan, Wen Shen, Yao Wu, Weiping Tu, Xiangdong Fang

**Affiliations:** 1 Department of Nephrology, The Second Affiliated Hospital of Nanchang University, No. 1, Minde Road, Nanchang, 330006 Jiangx People’s Republic of China; 2 Department of Cardiovascular Medicine, The Second Affiliated Hospital to Nanchang University, Nanchang, 330006 China

**Keywords:** VEGFA, PLA2R-associated idiopathic membranous nephropathy, PI3K/AKT signalling, Bioinformatics

## Abstract

**Background:**

The M-type phospholipase A2 receptor (PLA2R)-associated idiopathic membranous nephropathy (IMN) is a common immune-related disease in adults. Vascular endothelial growth factor A (VEGFA) is the key mediator of angiogenesis, which leads to numerous kidney diseases. However, the role of VEGFA in IMN is poorly understood.

**Methods:**

In the present study, we downloaded the microarray data GSE115857 from Gene Expression Omnibus (GEO). The differentially expressed genes (DEGs) were identified with R software. The cytoHubba plug-in were used to identify hub genes from the protein–protein interaction network. Gene set enrichment analysis (GSEA) was used to identify signalling pathway in IMN. CCK8 was performed to assess the cell viability in human vascular endothelial cells (HVECs). Then, passive Heymann nephritis (PHN) was induced in rats by a single tail vein injection of anti-Fx1A antiserum. Animals treated with VEGFA inhibitor bevacizumab (BV), with saline as a positive control. Proteinuria was evaluated by biochemical measurements. Immunohistochemistry and immunofluorescence was used to evaluate relative proteins expression. Electron microscopy was performed to observe the thickness of the glomerular basement membrane (GBM).

**Results:**

We revealed 3 hub genes, including one up-regulated gene VEGFA and two down-regulated genes JUN and FOS, which are closely related to the development of PLA2R-associated IMN. Pathway enrichment analysis found that the biological process induced by VEGFA is associated with PI3K/Akt signalling. GSEA showed that the signalling pathway of DEGs in GSE115857 was focused on angiogenesis, in which VEGFA acts as a core gene. We confirmed the high expression of VEGFA, PI3K, and AKT in IMN renal biopsy samples with immunohistochemistry. In HVECs, we found that BV suppresses cell viability in a time and dose dependent manner. *In vivo*, we found low dose of BV attenuates proteinuria via inhibiting VEGFA/PI3K/AKT signalling. Meanwhile, low dose of BV alleviates the thickening of the GBM.

**Conclusion:**

VEGFA/PI3K/AKT signalling may play significant roles in the pathogenesis of IMN, which may provide new targets for the treatment of IMN.

**Supplementary Information:**

The online version contains supplementary material available at 10.1186/s12882-022-02936-y.

## Introduction

Membranous nephropathy (MN) is one of the most common causes of nephritic syndrome in adults and is the second most common glomerulopathy to progress to end-stage renal disease (ESRD). Most cases are idiopathic (IMN), but ~ 20% are secondary to various causes, including cancers, infections, autoimmune diseases, and medications. The M-type phospholipase A2 receptor (PLA2R), thrombospondin type 1 domain-containing 7A (TSHD7A), and neural epidermal growth factor-like 1 protein (NELL-1) are major autoantigens against podocyte antigens in idiopathic membranous nephropathy (IMN) [1]. PLA2R-associated IMN is a primary membranous nephropathy with positive serum anti-PLA2R antibody, which covers 70% of IMN [2]. Circulating serum autoantibodies against the M-type phospholipase A2 receptor (PLA2R-AB) are a key biomarker in the diagnosis and monitoring of IMN [3]. Moreover, circulating PLA2R-AB is detectable months to years before documented non-nephrotic range proteinuria and biopsy-proven diagnosis in patients with MN [4]. Thus, the immune complex (IC) formed by PLA2R and serum autoantibody deposition on the sub-epithelial side of the glomerular basement membrane resulting in podocyte injury is the characteristic feature of IMN, in which membrane attack complex C5b-9 plays an essential role by inducing a variety of downstream pathways, including protein kinases, lipid metabolism, reactive oxygen species, growth factors/gene transcription, endoplasmic reticulum stress, and the ubiquitin–proteasome system [5, 6].

Vascular endothelial growth factor A (VEGFA), a homodimeric vasoactive glycoprotein, is the key mediator of angiogenesis. Angiogenesis, the formation of new blood vessels, is responsible for a wide variety of physio/pathological processes in kidney diseases, such as diabetic kidney disease and polycystic kidney disease [7]. In the renal glomeruli, VEGFA is mainly expressed in and secreted from podocytes and tubular epithelial cells, which induces renal injury through binding to the receptor VEGFR, mainly VEGFR2, which is expressed on the surface of endothelial cells [8]. In recent decades, studies that investigated dysregulation of VEGFA expression during kidney diseases have led to the identification of a crucial role of this proangiogenic factor in the renal capillary network [9, 10]. Ayumi Matsumoto et al. reported that VEGFA may be important for TSHD7A-associated IMN pathogenesis [11]. However, the role of VEGFA in PLA2R-associated IMN is poorly studied.

In the present study, we attempted to identify crucial genes and pathways that are involved in the pathogenesis of PLA2R-associated IMN. These results may provide new targets for the treatment of IMN.

## Materials and methods

### Ethics statement

In the study, 18 rats, 8 biopsies from humans with IMN, and 2 controls obtained from living donors were used.

All animal experimental procedures in this study were approved by the Ethics Committee of The Second Affiliated Hospital of Nanchang University (Approval number: SYXK (赣) 2010–0001), performed in the Molecular Center Lab of The Second Affiliated Hospital of Nanchang University, and implemented in line with the 8th edition of Guidelines for the Care and Use of Laboratory Animals published by the National Institutes of Health in 2011. A variety of laboratory procedures were performed to reduce the pain of rats or rabbits, such as heating pads, disinfection and replenishing liquid with normal saline.

All cases used in this study were defined by a kidney biopsy diagnosis of idiopathic MN, and any suspected secondary cases due to drugs, malignancy, infection, or autoimmune disease were excluded. All patients’ consents were obtained and approved by the Ethics Committee of The Second Affiliated Hospital of Nanchang University. The study protocol was reviewed and approved by an institutional review board.

### Microarray data information

We used “membranous nephropathy” as the keyword to search the Gene Expression Omnibus (GEO) database. We selected the expression profiling of the array: the attribute name was tissue, the organisms were Homo sapiens, and only the array with high expression of M-type phospholipase A2 receptor 1 (PLA2R1) was chosen for further analysis (Accession number: GSE115857). GSE115857 contains 11 renal biopsies from IMN patients and 7 renal biopsies from living donors, which is based on the GPL14951 (Illumina HumanHT-12 WG-DASL V4.0 R2 expression beadchip) platform.

Because the data in this experiment were all from public databases, there was no need for approval by the ethics committee.

### Identification of differentially expressed genes

The limma package in R software (Version 3.6.2) was utilized to screen differentially expressed genes (DEGs) between the IMN group and living donor group. An adjusted *p* value of < 0.05 and |logFC|> 1.0 were considered statistically significant. The ggplot2 and RColorBrewer packages in R were used to plot the volcano map and the heatmap in the two groups.

### Gene ontology and pathway enrichment analysis

Gene Ontology (GO) includes three categories: molecular function (MF), biological process (BP), and cellular component (CC). GO and KEGG pathway analyses were performed via the Database for annotation [12–14], Visualization and Integrated Discovery (DAVID) [15] and Cytoscape ClueGo plug-in. Gene set enrichment analysis (GSEA) determines whether an a priori defined set of genes has statistically significant differences in expression under two different biological conditions [16]. This analysis was performed using GSEA software 4.0.3 from the Broad Institute. The gene set “h.all.v7.1.symbols.gmt”, which summarizes and represents specific, well-defined biological states or processes, was downloaded from the Molecular Signatures Database (http://software.broadinstitute.org/gsea/msigdb/index.jsp). The normalized enrichment score (NES) was determined by analysis of 1000 permutations. A gene set was considered significantly enriched when the P value was less than 0.05 and the false discovery rate (FDR) was less than 0.25.

### Protein–protein interaction network and hub gene identification

To evaluate the interactive relationships among DEGs, the STRING online database (https://string-db.org) was used [17]. The protein–protein interaction (PPI) network of DEGs was visualized with Cytoscape software, and central genes were identified by cytoHubba to select the hub genes, which were intersected by the betweenness, bottleneck, closeness, and degree methods [18]. Significant modules in the PPI network were identified by Molecular Complex Detection (MCODE), a plug-in of Cytoscape software that clusters a network based on topology to recognize closely connected regions [19]. The MCODE algorithm sorts and identifies each identified module. The higher the score, the stronger the gene association in this module. The parameters of DEG clustering and scoring were set as follows: MCODE score ≥ 4, degree cut-off = 2, node score cut-off = 0.2, max depth = 100 and k-score = 4. Pathway and BP enrichment analyses were performed for DEGs in the identified modules using ClueGo, a plug-in of Cytoscape software.

### Detection of serum anti-PLA2R and THSD7A levels by enzyme-linked immunosorbent assay

Blood samples were collected after renal biopsy pathology results were obtained from Jan 2020 to March 2020 in Nephrology Department of Nanchang University Second Affiliated Hospital. The concentrations of serum anti-PLA2R and THSD7A were evaluated using enzyme-linked immunosorbent assay (ELISA) kits according to the manufacturer’s instructions, which were all purchased from Jiangxi Oumeng Biotechnology.

### Experimental animal

Male Wistar rats, Sprague–Dawley (SD) rats, and New Zealand white rabbits were obtained from the Animal Center of Nanchang University (Nanchang, China). All rats were housed under standard specific pathogen-free (SPF) conditions with a 12 h light/dark cycle at 22 ~ 24 °C and allowed to eat a standard diet and drink ad libitum.All animals’ health and behaviour were monitored daily. In the present study, the rabbits were housed in a clean-grade animal lab. Rabbits were anaesthetized with isoflurane inhalation (a concentration of 2 to 3% was used for induction and 1.5 to 2% for maintenance) before the experiment. All rat and rabbit experiments took place at the Animal Center of Nanchang University between May 2021 and October 2021.. After the experiment, carbon dioxide was asphyxiated and supplied at a flow rate of 20% (5 L/min) cage volume per minute for euthanasia in rabbits. If necessary, the animals were euthanized by intraperitoneal injection of 1% sodium pentobarbital (100 mg/kg). The death of experimental animals can be determined by touching their heartbeat and observing their pupils.

### CCK8 assay

Immortalized human vascular endothelial cells (HVECs) were purchased from ScienCell (San Diego, CA, USA) and cultured under standard cell culture conditions at 37 °C and 5% CO2 in a humidified incubator using endothelial cell basal medium (ECM) (Pierce HyClone, Fremont, CA, USA) supplemented with 100 U/mL penicillin, 100 μg/ml streptomycin, 10% (v/v) foetal bovine serum and 1% endothelial cell growth factor.

After being stimulated with different concentrations of bevacizumab (BV) for different times, we removed the cell culture medium and washed the cells twice with PBS. Then, 100 µl cell culture medium supplemented with 10 µl CCK-8 solution (C0038, Beyotime, China) was added to the cells and incubated for 2 h. Finally, we detected the OD600 to calculate the relative cell viability.

### Establishment of the passive Heymann nephritis model and treatment

The passive Heymann nephritis (PHN) model was used to mimic human MN. The PHN model was prepared with a standard protocol [20]. Briefly, the Fx1A antigen was acquired from the renal cortices of Wistar rats. Then, male New Zealand white rabbits were immunized with Fx1A antigen, and rabbit antiserum was prepared. PHN was induced in 12 male SD rats (8 weeks old) with body weights of 250 ~ 280 g through a single intraperitoneal injection of anti-Fx1A antiserum (6 mL/kg body weight) for 12 weeks. The SD rats were randomly divided based on the proteinuria level at one week after anti-Fx1 infusion. Then, the selected PHN rats were randomly divided into the following two groups (6 rats/group): the model group and the VEGFA inhibitor bevacizumab (BV) group. In addition, 6 of the healthy SD rats (8 weeks old) were selected as normal controls. The VEGFA inhibitor group rats received a monoclonal antibody against VEGFA and bevacizumab (BV) intraperitoneally twice a week for 30 days. On day 30, the rats were placed individually in metabolic cages (Jeungdo Bio & Plant Co., Seoul, Korea) for 24 h to collect urine. The volume of urine samples was recorded and then centrifuged at 1000 rpm for 10 min, and the supernatants were stored at -20 °C until analysis. The next day, rats were all euthanized after being fasted for 12 h with pentobarbital sodium (100 mg/kg), and blood samples were collected from the abdominal aorta and allowed to clot at room temperature, followed by centrifugation at 3000 rpm for 15 min to collect serum, and then stored at -80 °C for biochemical assays. After that, the kidney of each rat was immediately removed. The right kidney was fixed in 4% paraformaldehyde for histopathological studies, and the other kidney was stored at -80 °C for biochemical analysis.

### Immunohistochemistry

PI3K (mouse anti-mouse anti-PI3 kinase antibody, 1:150, ab140307, Abeam, UK), AKT (rabbit anti-rabbit anti-AKT1 + AKT2 + AKT3 antibody, 1:250, ab179463, Abeam, UK), and VEGFA (rabbit anti-rabbit anti-VEGF antibody, 1:20000, ab39250, Abcam, UK) were used in immunohistochemistry.

We selected 10 renal biopsy samples, including 8 IMN and 2 normal renal tissue samples (Supplementary Table 1). Serum anti-PLA2R levels were significantly increased in 8 patients with IMN.

Renal tissue samples were fixed in 4% paraformaldehyde, rinsed with tap water, stained with a SABC immunohistochemistry kit, developed with diaminobenzidine (DAB), dehydrated with a graded series of ethanol, cleared with xylene and sealed with neutral gum. Finally, the renal sections were observed under a 200 × optical microscope (Olympus, Japan). Quantification of target protein staining was performed only on glomeruli using an IHC profiler in Image-Pro Plus, in which the intensity of the staining was quantified.

### Immunofluorescence

Renal tissue was fixed with 4% paraformaldehyde, embedded in paraffin and cut into 5-μm-thick slices. The slice was processed with dewaxing, gradient alcohol dehydration, antigen repair and washing in phosphate-buffered saline (PBS) three times. For immunofluorescence, slides were incubated with VEGFA overnight at 4 °C. Then, their corresponding anti-FITC-conjugated secondary antibody was incubated with slices for 1 h at room temperature in the dark. Finally, images were captured using a fluorescence microscope.

### Electron microscopy analysis

Kidney tissues fixed in 2.5% glutaraldehyde were post‑fixed in 1% osmium tetroxide at 4 °C for 2 h. After dehydration, the samples were embedded in EMbed 812 resin (Thermo Fisher Scientific, Inc., Waltham, MA, USA) and polymerized at 60 °C for 48 h. The ultrathin sections (60–80 nm) were cut and stained with 1% uranyl acetate for 10 min, followed by 2% lead citrate buffer for 2 min at 37 °C. The structure of the glomerulus was observed under a Hitachi High-Tech7700 electron microscope.

### Statistical analysis

Data are expressed as the mean ± standard deviation (SD). Comparisons between two groups were made by rank sum test. One-way analysis of variance followed Tukey’s test was performed for comparisons between multiple groups. *p* < 0.05 was considered statistically significant.

## Results

### Identification of DEGs

**Fig.1:**
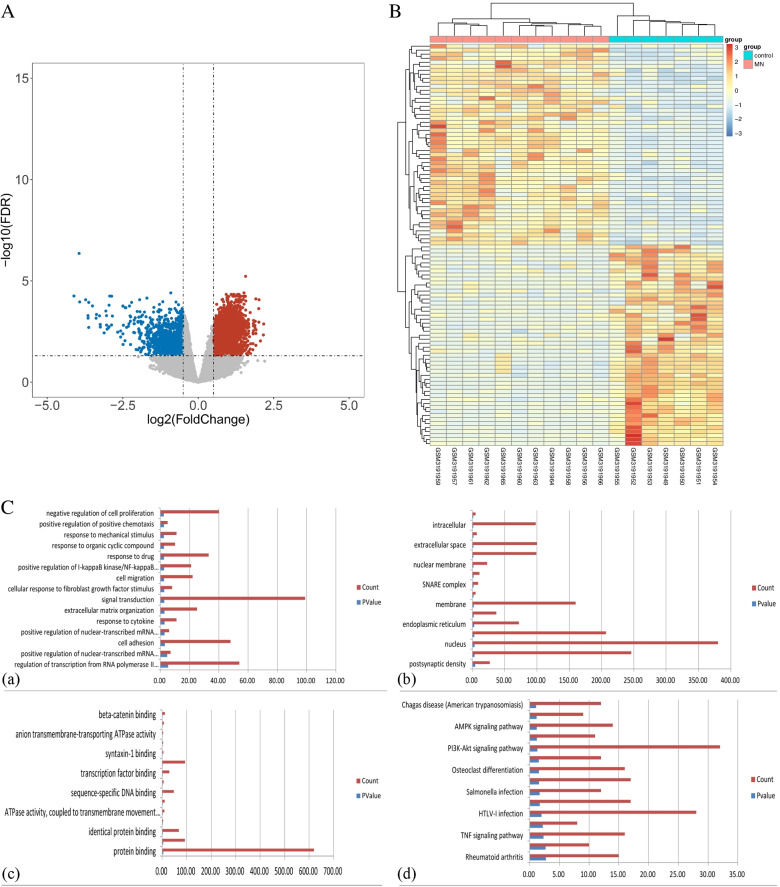
**A** Volcano plot of DEGs in IMN. The cut-off criteria were |log2Fc|> 1 and *P* < 0.05. The red dots represent the up-regulated genes, and the blue dots denote the down-regulated genes. The grey dots indicate the genes with |log2Fc|< 1 and/or *P* > 0.05. **B** Heatmap of the top 50 up-regulated genes and the top 50 down-regulated genes. Grey indicates relatively low expression, and red indicates relatively high expression. **C** GO enrichment and KEGG pathway analysis of DEGs: (a) Biological process. (b) Cellular component. (c) Molecular function. (d) KEGG pathway

A total of 1422 DEGs were identified in GSE115857, including 952 up-regulated genes and 470 down-regulated genes. The volcano plot of all DEGs and heatmap of the top 50 up-regulated genes and the top 50 down-regulated genes are shown in Fig. 1A and B, respectively.

### Pathway and GO enrichment analysis of DEGs

GO enrichment and KEGG pathway analysis were performed with DAVID, which consists of three terms, as shown in Fig. 1C(a), (b), (c), and (d).

The top 15 biological process (BP) of DEGs in GSE115857 was clustered in regulation of transcription from RNA polymerase II promoter (GO:0006357, 54DEGs), positive regulation of nuclear-transcribed mRNA poly(A) tail shortening (GO:0060213, 7DEGs), cell adhesion (GO:0007155, 48DEGs), positive regulation of nuclear-transcribed mRNA catabolic process, deadenylation-dependent decay (GO:1900153, 6DEGs), response to cytokine (GO:0034097,11DEGs), extracellular matrix organization (GO:0030198,25DEGs), signal transduction (GO:0007165,99DEGs), cellular response to fibroblast growth factor stimulus (GO:0044344, 8DEGs), cell migration (GO:0016477,22DEGs), positive regulation of I-kappa B kinase/NF-kappa B signalling (GO:0043123,21DEGs), response to drug (GO:0042493,33DEGs), response to organic cyclic compound (GO:0014070,10DEGs), response to mechanical stimulus (GO:0009612,11DEGs), positive regulation of positive chemotaxis (GO:0050927,5DEGs), and negative regulation of cell proliferation (GO:0008285,40DEGs) (Supplement Table 2).

The top 15 cellular component (CC) of DEGs in GSE115857 was clustered in postsynaptic density (GO:0014069,27DEGs), cytosol (GO:0005829,246DEGs), nucleus (GO:0005634,380DEGs), nucleoplasm (GO:0005654,207DEGs), endoplasmic reticulum (GO:0005783,72DEGs), focal adhesion (GO:0005925,37DEGs), membrane (GO:0016020,160DEGs), CCR4-NOT complex (GO:0030014,5DEGs), SNARE complex (GO:0031201,9DEGs), cell projection (GO:0042995,11DEGs), nuclear membrane (GO:0031965,23DEGs), mitochondrion (GO:0005739,99DEGs), extracellular space (GO:0005615,100DEGs), phagocytic vesicle (GO:0045335,7DEGs), intracellular (GO:0005622,98DEGs), and mast cell granule (GO:0042629,5DEGs).

The top 15 molecular function (MF) of DEGs in GSE115857 was clustered in protein binding (GO:0005515,620DEGs), transcription factor activity, sequence-specific DNA binding (GO:0003700,92DEGs), identical protein binding (GO:0042802,68DEGs), neurotrophin TRKA receptor binding (GO:0005168,4DEGs), ATPase activity, coupled to transmembrane movement of substances (GO:0042626,9DEGs), steroid hormone receptor activity (GO:0003707,10DEGs), sequence-specific DNA binding (GO:0043565,47DEGs), transcription factor activity, RNA polymerase II core promoter proximal region sequence-specific binding (GO:0000982, 6DEGs), transcription factor binding (GO:0008134,29DEGs), zinc ion binding (GO:0008270,93DEGs), syntaxin-1 binding (GO:0017075,5DEGs), palmitoyl-CoA hydrolase activity (GO:0016290,4DEGs), anion transmembrane-transporting ATPase activity (GO:0043225,4DEGs), RNA polymerase II activating transcription factor binding (GO:0001102,7DEGs), and beta-catenin binding (GO:0008013,11DEGs).

The top 15 Kyoto Encyclopedia of Genes and Genomes (KEGG) pathway of DEGs in GSE115857 was clustered in Rheumatoid arthritis (hsa05323,15DEGs), ABC transporters (hsa02010,10DEGs), TNF signalling pathway (hsa04668,16DEGs), SNARE interactions in vesicular transport (hsa04130,8DEGs), HTLV-I infection (hsa05166,28DEGs), Ubiquitin mediated proteolysis (hsa04120,17DEGs), Salmonella infection (hsa05132,12DEGs), Cell adhesion molecules (CAMs) (hsa04514,17DEGs), Osteoclast differentiation (hsa04380,16DEGs), NF-kappa B signalling pathway (hsa04064,12DEGs), PI3K-Akt signalling pathway (hsa04151,32DEGs), ECM-receptor interaction (hsa04512,11DEGs), AMPK signalling pathway (hsa04152,14DEGs), Amphetamine addiction (hsa05031,9DEGs), and Chagas disease (American trypanosomiasis) (hsa05142,12DEGs) (Supplement Table 3).

### PPI network and hub gene analysis

**Fig. 2:**
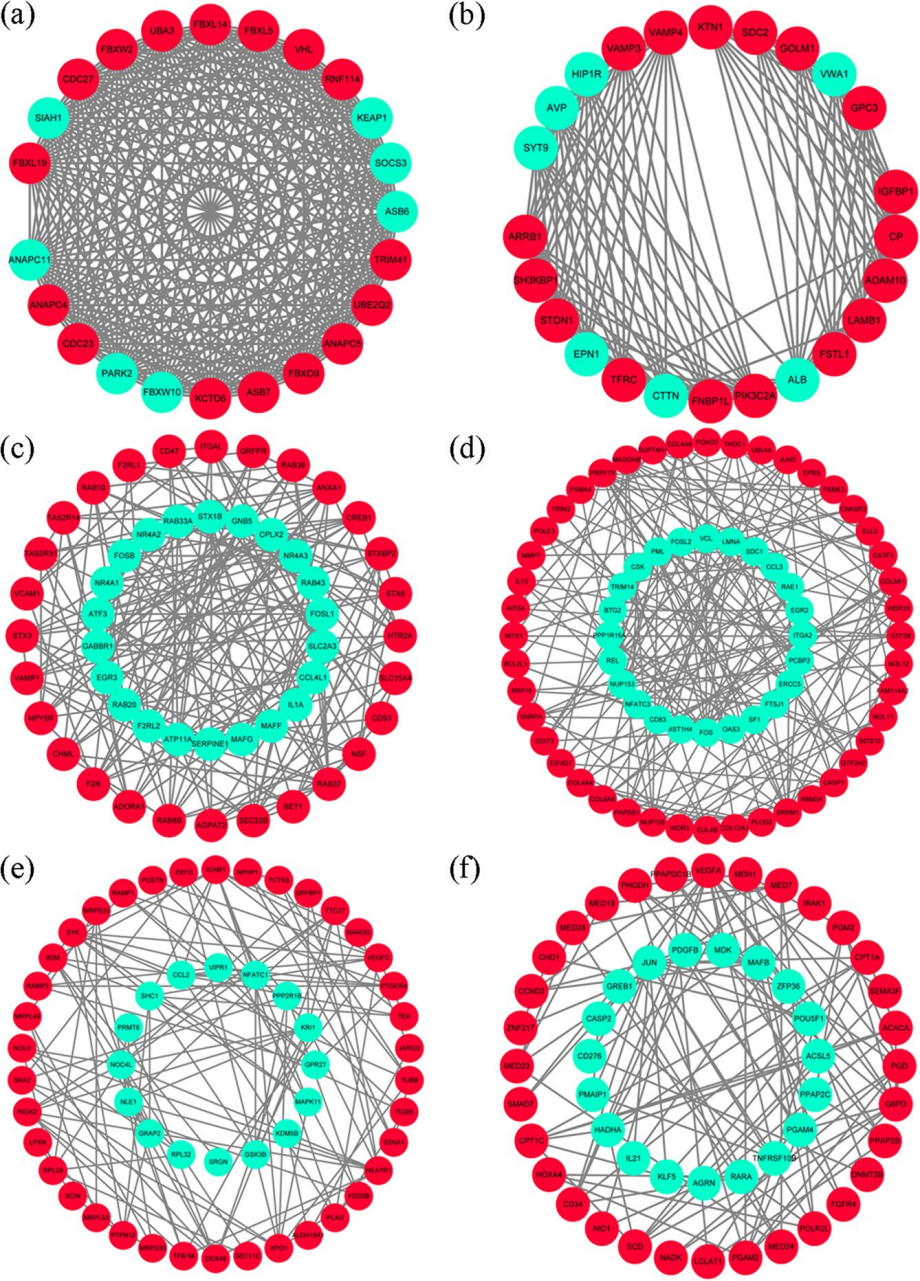
Six modules identified by MCODE: 6 modules from the PPI network were selected. The red nodes represent the up-regulated genes, and the green nodes represent the down-regulated genes: (**a**) module 1; (**b**) module 2; (**c**) module 3; (**d**) module 4; (**e**) module 5; (**f**) module 6

To explore the relationship between these DEGs and to identify hub genes, a PPI network of DEGs was constructed using the STRING online database and visualized using Cytoscape. There were 1199 nodes and 5490 edges in the PPI network, including 760 up-regulated genes and 350 down-regulated genes. In addition, 6 modules from the PPI network were selected using the MCODE plug-in of Cytoscape: module 1 (score = 24), consisting of 24 nodes and 276 edges (Fig. 2 (a)), module 2 (score = 13.04), consisting of 26 nodes and 163 edges (Fig. 2 (b)), module 3 (score = 8.078), consisting of 52 nodes and 206 edges (Fig. 2 (c)), module 4 (score = 5.971), consisting of 71 nodes and 209 edges (Fig. 2 (d)), module 5 (score = 5.051), consisting of 60 nodes and 149 edges (Fig. 2 (e)), and module 6 (score = 4.654), consisting of 53 nodes and 121 edges (Fig. 2(f)). Then, GO and pathway enrichment analyses of these module genes were performed by the CluGo plug-in of Cytoscape.

For the BP, genes from module 1 were most significantly clustered in positive regulation of mitotic metaphase/anaphase transition (GO:0045842, 5DEGs), there is no BP enrichment in module 2, genes from module 3 were most significantly clustered in organelle fusion (GO:0048284, 15DEGs) and regulation of glomerular filtration (GO:0003093, 4DEGs), genes from module 4 were most significantly clustered in ribonucleoprotein complex export from nucleus (GO:0071426, 10DEGs), genes from module 5 were most significantly clustered in cell adhesion mediated by integrin (GO:0033627, 8DEGs) and maturation small subunit ribosomal RNA (SSU-rRNA) (GO:0030490, 6DEGs), genes from module 6 were most significantly clustered in pentose-phosphate shunt, oxidative branch (GO:0009051, 4DEGs) (Supplementary Fig. 1, Supplement Table 4).

For CC, genes from module 1 were most significantly clustered in cullin-RING ubiquitin ligase complex (GO:0031461, 12DEGs), genes from module 2 were most significantly clustered in clathrin-coated vesicle (GO:0030136, 13DEGs), genes from module 3 were most significantly clustered in SNARE complex (GO:0031201, 9DEGs), specific granule (GO:0042581, 15DEGs), phagocytic vesicle (GO:0045335, 11DEGs), and mast cell granule (GO:0042629, 4DEGs) (Supplementary Fig. 2).

For MF, there was no MF enrichment in module 1. Genes from module 2 were most significantly clustered in clathrin adaptor activity (GO:0035615, 3DEGs), genes from module 3 were most significantly clustered in SNAP receptor activity (GO:0005484, 7DEGs), glucocorticoid receptor binding (GO:0035259, 3DEGs), and thrombin-activated receptor activity (GO:0015057, 3DEGs), genes from module 4 were most significantly clustered in extracellular matrix structural constituent conferring tensile strength (GO:0030020, 5DEGs), and genes from module 6 were most significantly clustered in phosphatidate phosphatase activity (GO:0008195, 3DEGs) (Supplementary Fig. 3).

For KEGG enrichment, genes from module 1 were clustered in ubiquitin mediated proteolysis (KEGG:04120, 15DEGs), genes from module 3 were clustered in soluble NSF Attachment Protein Receptor (SNARE) interactions in vesicular transport (KEGG:04130, 8DEGs), genes from module 4 were clustered in extracellular matrix (ECM)-receptor interaction (KEGG:04512, 8DEGs), there is no KEGG enrichment in module 2,5 and 6 (Supplementary Fig. 4, Supplement Table 5).

**Fig. 3:**
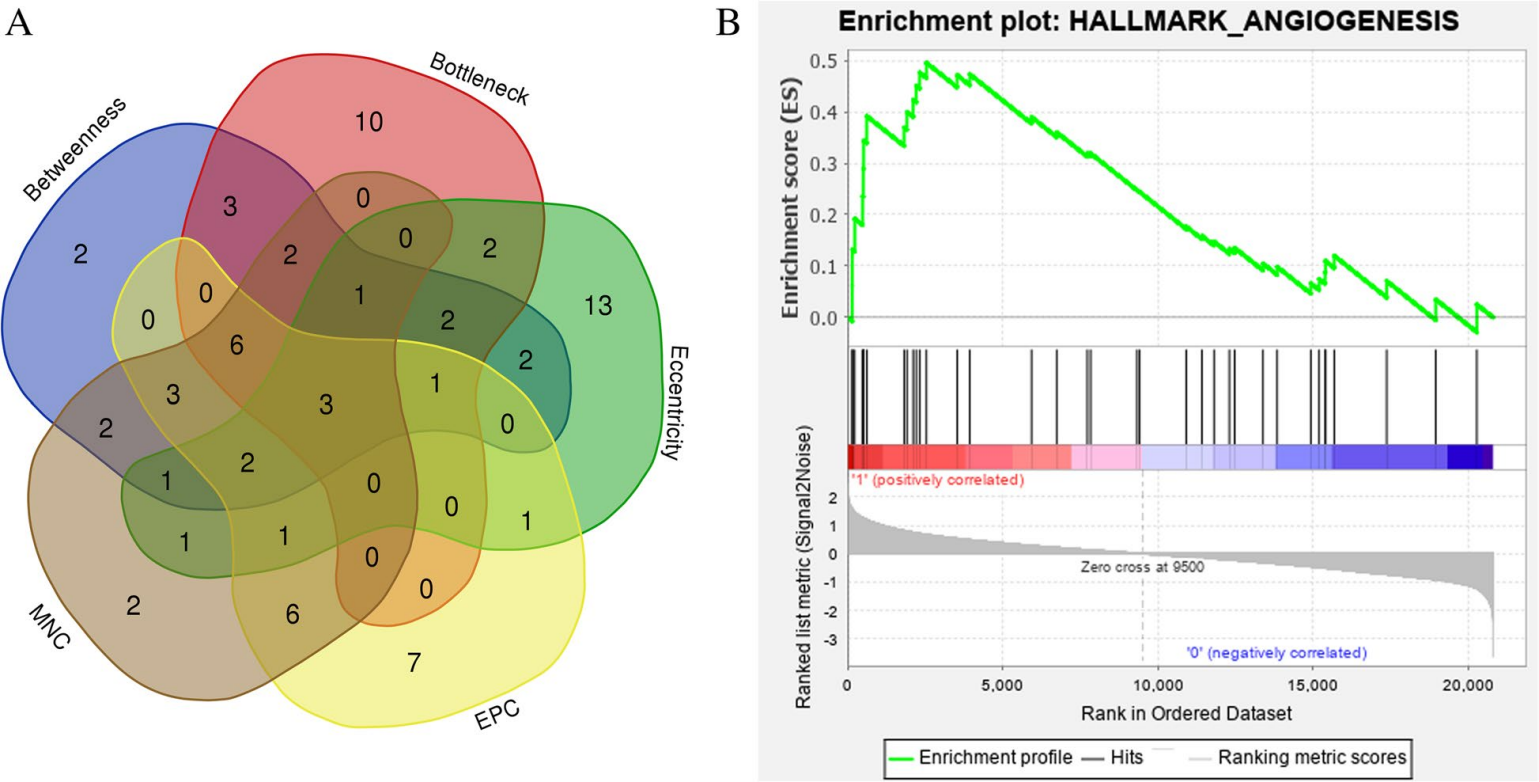
**A** The hub genes in the PPI network screened out via intersected by Betweenness, Bottleneck, Eccentricity, EPC, and MNC methods. **B** Signalling pathway activated in kidney biopsies from IMN patients using GSEA

In the present study, we used cytoHubba to choose hub genes. According to the five classification methods in cytoHubba, the top 30 hub genes selected by these ranked methods in cytoHubba are shown in Supplement Table 6. Finally, three central genes were identified by overlapping the first 30 genes, as shown in Fig. 3A. VEGFA was the best central gene based on the five ranked methods. JUN and FOS were also selected as hub genes.

### GSEA identifies signalling pathways in IMN

We compared the data sets for IMN and living donors using GSEA to identify signalling pathways. The results indicated significant differences (FDR < 0.25, NOM *P* value < 0.05) in the enrichment of the MSigDB collection (h.all.v7.1.symbols.gmt). We selected the most significantly enriched signalling pathways based on the normalized enrichment score (NES) (Fig. 3B, Supplement Table 7). The results indicated that the data set with IMN was enriched for angiogenesis. Thirteen core genes were found, including PTK2, SLCO2A1, COL3A1, FSTL1, CCND2, SERPINA5, VEGFA, VAV2, OLR1, APP, POSTN, VCAN, and NRP1 (Supplementary Fig. 5).

### Immunohistochemistry

**Fig. 4:**
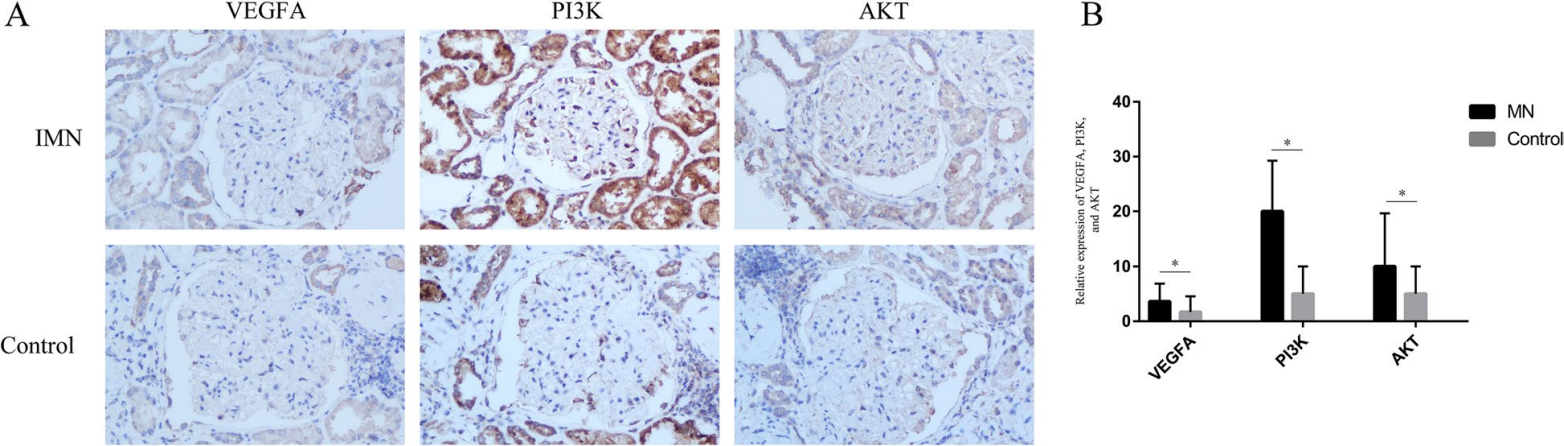
The relative expression of VEGFA, PI3K, and AKT. **A** Immunohistochemistry; **B** The relative expression of VEGFA, PI3K, and AKT. ^*^p < 0.05, compared with control group

In this study, we examined the expression levels of VEGFA, PI3K and AKT in glomeruli from IMN patients and healthy controls using immunohistochemical staining. An average of 5.2 glomeruli were quantified per patient. Compared with the control group, the contents of VEGFA, PI3K and AKT were significantly increased in IMN patients and had weak positive expression (*p* < 0.05) (Fig. 4A and B). These results show that VEGFA may promote the occurrence of PLA2R-associated IMN by stimulating angiogenesis via PI3K/AKT signalling.

## VEGFA inhibitor alleviates endothelial injury in an *in vivo* study

**Fig. 5:**
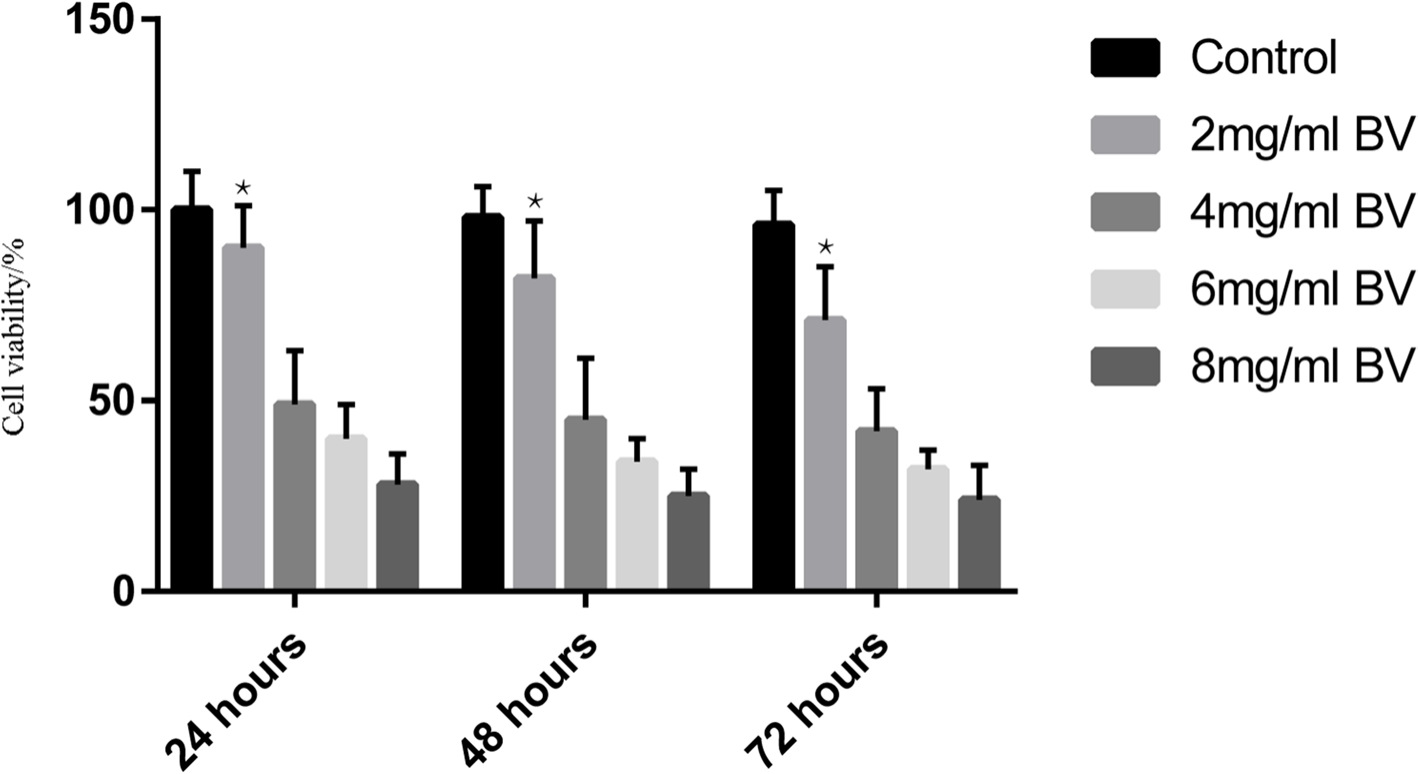
Bevacizumab (BV) downregulates the viability of human vascular endothelial cells (HVECs) in a dose-dependent manner

**Fig. 6:**
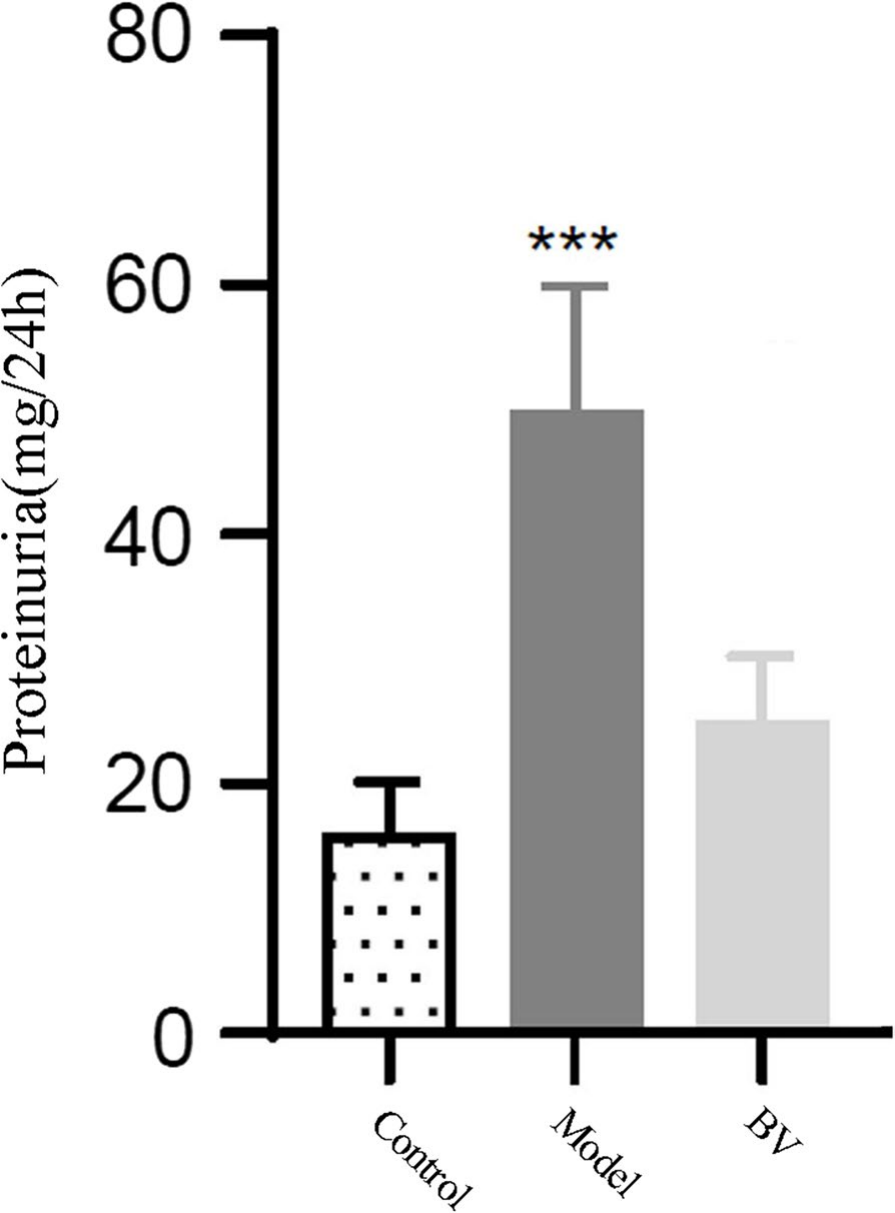
Bevacizumab (BV) decreased proteinuria excretion compared with the model group (*p* < 0.05)

**Fig. 7:**
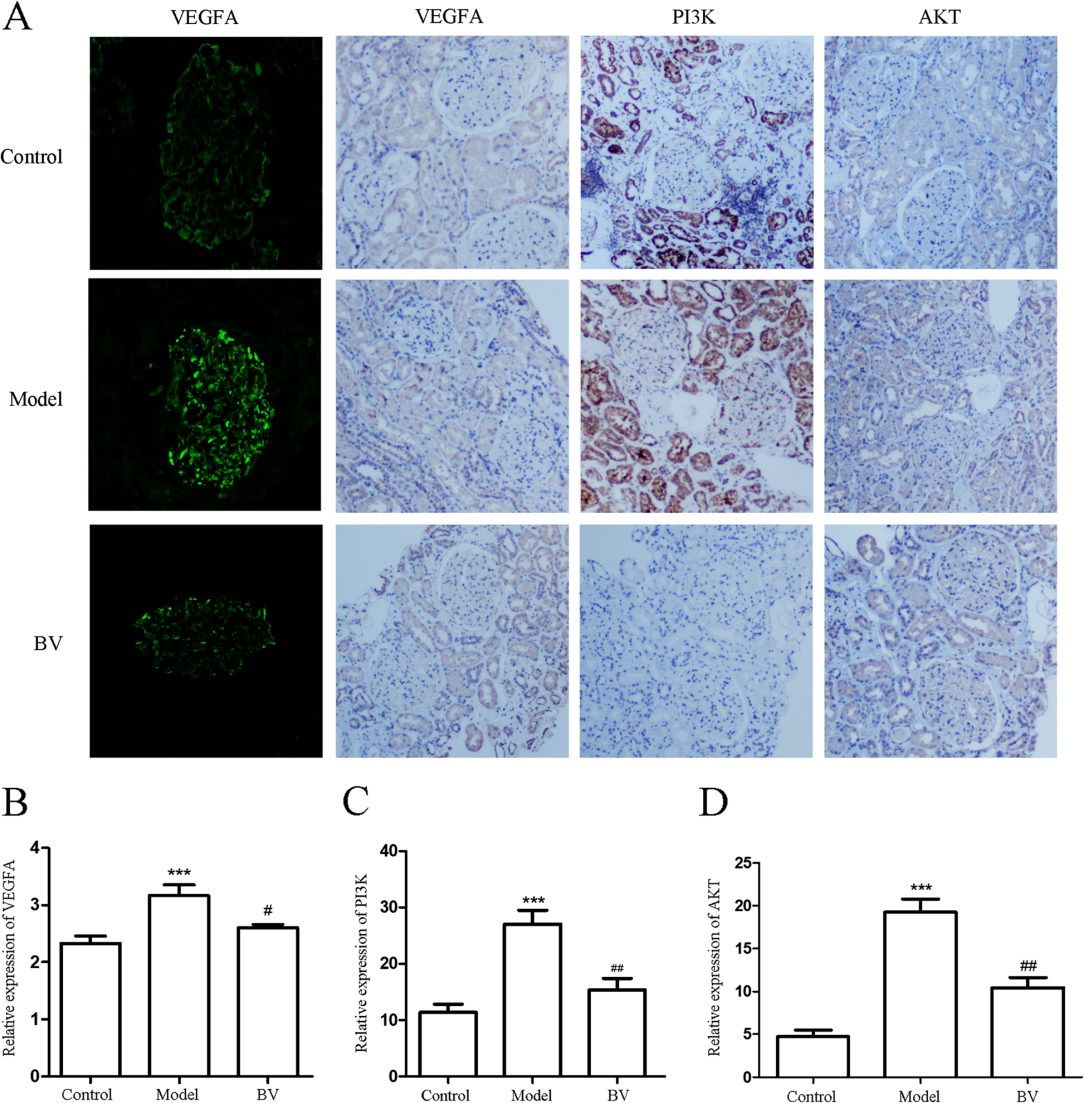
**A** Immunofluorescence and immunohistochemistry showed that the expression of VEGFA in the BV group was alleviated and accompanied by suppression of PI3K and AKT compared with the model group. **B** Immunohistochemistry data showed that the expression of VEGFA in the BV group was alleviated compared with that in the model group (*p* < 0.05). **C** Immunohistochemistry data showed that the expression of PI3K in the BV group was decreased compared with that in the model group (*p* < 0.05). **D** Immunohistochemistry data showed that the expression of AKT in the BV group was alleviated compared with that in the model group (*p* < 0.05)

**Fig. 8:**
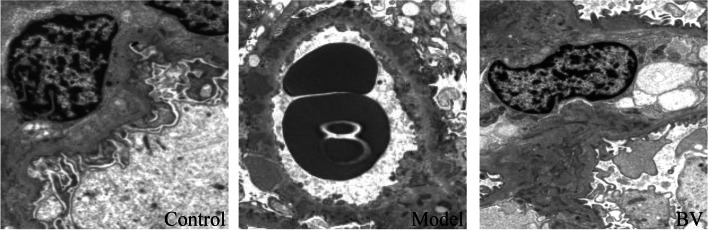
Electron microscopy showed that the glomerular basement membrane and the morphology of glomerular endothelial cells were alleviated in the VEGFA inhibitor group compared with the model group

First, we used a CCK-8 kit to evaluate cell viability after incubation with different concentrations (2 mg/ml, 4 mg/ml, 6 mg/ml and 8 mg/ml) of BV for different times (24 h, 48 h, and 72 h), and saline was used as a control. We found that the higher the concentration of BV added was, the lower the cell viability; the longer the high BV incubation time was, the lower the cell viability (Fig. 5). Thus, we chose 2 mg/kg BV as stimuli in PHN rats. *In vitro* experiments demonstrated that the VEGFA inhibitor BV decreased proteinuria excretion compared with the model group (*p* < 0.05) (Fig. 6). Moreover, immunofluorescence and immunohistochemistry showed that the expression of VEGFA in the BV group was alleviated and accompanied by suppression of PI3K and AKT compared with the model group (Fig. 7A). Immunohistochemistry data showed that along with the decreased expression of VEGFA in the VEGFA inhibitor group, the expression of PI3K and AKT was obviously decreased compared with that in the model group (*p* < 0.05) (Fig. 7B, C, and D). Furthermore, the glomerular basement membrane and the morphology of glomerular endothelial cells were alleviated in the VEGFA inhibitor group compared with the model group, as shown by electron microscopy (Fig. 8). All data suggested that a VEGFA inhibitor may alleviate MN by decreasing the expression of PI3K and AKT.

## Discussion

In recent years, with the use of vascular endothelial growth factor A (VEGFA) inhibitors in oncology, the number of patients with kidney disease has increased, mainly manifesting as thrombotic microangiopathy, minimal change nephropathy, and collapsing focal segmental glomerulosclerosis [21, 22]. Moreover, rituximab, a B cell-targeted monoclonal antibody that has become the first-line therapy in IMN according to KDIGO CLINICAL PRACTICE GUIDELINE ON GLOMERULAR DISEASES 2021 [23], showed its ability to decrease the VEGFA level in plasma in patients with recurrent mantle cell lymphoma [24]. Additionally, Ayumi Matsumoto et al. reported that VEGFA may be important for TSHD7A-associated IMN pathogenesis [11]. These data suggest that VEGFA may be involved in the development of MN. In this study, we identified 1422 genes as DEGs in kidneys from IMN patients compared to living donors, 3 hub genes, one up-regulated gene VEGFA, two down-regulated genes JUN and FOS, and six significant modules selected from a PPI network, which partially revealed the molecular mechanism in IMN and may be used to develop novel targets for IMN treatment. We validated that the expression of VEGFA, PI3K and AKT was upregulated *in vivo*. Furthermore, we revealed that the VEGFA inhibitor bevacizumab plays a significant role in decreasing proteinuria and maintaining the integrity of glomerular endothelial cells via PI3K/AKT signalling *in vitro*. Thus, we concluded that VEGFA promotes the occurrence of PLA2R-associated IMN by stimulating angiogenesis via PI3K/AKT signalling. Moreover, we found that soluble *N*-ethylmaleimide–sensitive factor attachment protein receptor (SNARE) interactions in vesicular transport may be involved in the occurrence or progression of PLA2R-associated IMN.

Angiogenesis is a process of new blood vessel formation from existing vessels, which are essential for the formation of normal kidney structures and for the functions of glomeruli [25]. Abnormal capillary formation in the kidney may cause morphological changes in glomeruli, as well as infiltration of inflammatory cells [21, 26]. Angiogenesis is tightly regulated by a balance of proangiogenic and antiangiogenic factors, in which the most potent proangiogenic factor, vascular endothelial growth factor A (VEGFA), has been intensively investigated [21]. VEGFA is the predominant isoform of VEGF in humans and one of the most potent proangiogenic factors, which is mainly expressed in and secreted from podocytes [9] and renal tubular epithelial cells in glomeruli [7]. Expression of VEGFA promotes endothelial cell proliferation, migration, and survival but can also be associated with vascular hyperpermeability in inflammatory conditions, including inflammatory kidney diseases, by binding to the receptor VEGFR2 [27]. Neuropilin 1 (NRP1) functions as a coreceptor for VEGFA and is required for complete activation of VEGFR2 [28]. As VEGFR2 is expressed on the surface of endothelial cells, VEGFA-mediated epithelial–endothelial crosstalk in the glomeruli plays an essential role in numerous renal injuries [29]. Moreover, Gebran Khneizer et al. reported a case that describes self-limited biopsy-proven membranous nephropathy after intravitreal bevacizumab injections [30]. In the present study, we found that VEGFA and NRP1 are highly expressed in kidney tissue from IMN patients. GSEA confirmed that VEGFA up-regulation promotes angiogenesis in the IMN group. Pathway enrichment analysis found that the biological process induced by VEGFA is associated with PI3K/Akt signalling. Moreover, we validated that the expression of VEGFA, PI3K and AKT was upregulated *in vivo*. Furthermore, we revealed that the VEGFA inhibitor bevacizumab plays a significant role in decreasing proteinuria and maintaining the integrity of glomerular endothelial cells via PI3K/AKT signalling *in vitro*. In addition, the biological process of module 3 was clustered in cell migration involved in sprouting angiogenesis, which is a main type of angiogenesis. Thus, VEGFA activates PI3K/Akt signalling by binding to VEGFR2 to accelerate angiogenesis and leads to vascular hyperpermeability, which results in increased filtration of inflammatory factors, complement, and cytokines, eventually leading to immune complex (IC) and MAC formation. Similarly, a study reported that VEGFA may increase vascular permeability and promote inflammatory infiltrations causing glomerular disease in diabetes [31]. These results suggest that regulation of the VEGFA/PI3K/Akt signalling pathway may be a treatment strategy for IMN.

**Fig. 9:**
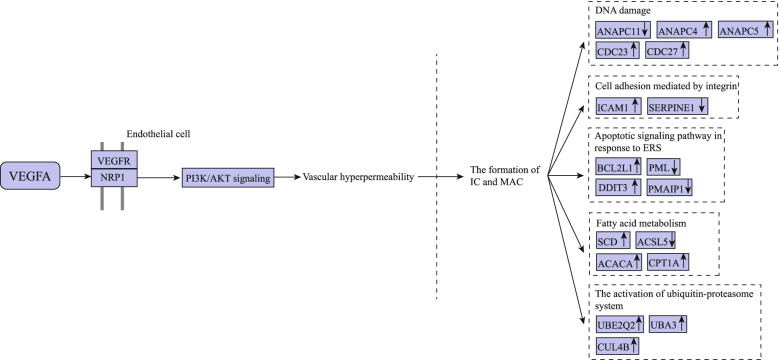
The possible mechanisms by which VEGFA/PI3K/Akt is involved in IMN

Complement-mediated cell injury has been demonstrated to contribute to IMN [5]. With complement activation, the formation of membrane attack complex C5b-9 in plasma membranes on the soles of the foot processes of visceral glomerular epithelial cells, which has been shown to induce kidney cell injury through activating a variety of downstream pathways, including focal adhesion kinases [32], lipid metabolism, reactive oxygen species, growth factors/gene transcription, endoplasmic reticulum stress, the ubiquitin–proteasome system [5] and DNA damage [33], impacts the integrity of the cytoskeleton and slit diaphragm proteins and contributes to the pathogenesis of mesangial-proliferative glomerulonephritis, thrombotic microangiopathy, and acute kidney injury [5]. In our study, six modules were found from the PPI network. Our finding remains consistent with previous studies. The biological process of genes from six modules were clustered in positive regulation of mitotic metaphase/anaphase transition, regulation of glomerular filtration, cell adhesion mediated by integrin, pentose-phosphate shunt, oxidative branch, intrinsic apoptotic signalling pathway in response to endoplasmic reticulum stress, nucleotide-excision repair, DNA incision, and fatty acid metabolism. The KEGG pathway enrichment was clustered in ubiquitin-mediated proteolysis and ECM-receptor interaction. Thus, it seems that the vascular hyperpermeability induced by VEGFA/PI3K/Akt signalling plays an initial role in the development of IMN (Fig. 9).

Notably, we found that SNARE interactions in vesicular transport were involved in the development of IMN, which was the first report in this paper. Soluble *N*-ethylmaleimide–sensitive factor attachment protein receptor (SNARE) proteins are essential for exocytosis, mediating the fusion of vesicles with their target membrane. A study reported that SNARE proteins mediate the fusion of autophagosomes with endolysosomal vesicles, which mediates autophagosome maturation, resulting in autophagy [34]. In addition, inhibiting autophagosome-induced excessive autophagy ameliorates proteinuria and protects against glomerular and podocyte injury in nephrotic syndrome rats [35]. Moreover, with the accumulation of autophagosomes, cell viability is altered, which directly induces cellular toxicity [36]. In our report, we found that genes encoding SNARE proteins, including STX6, STX3, VAMP1, VAMP3, VAMP4, STX1B, and SEC22B, are highly expressed in kidney biopsies from IMN patients, which means that inhibitors of the SNARE interaction may provide a new therapeutic strategy in treating IMN; however, this needs to be verified experimentally.

It should be noted that there were some limitations to our research. First, a recent study demonstrated that there are three non-HLA loci, namely, NFKB1, IRF4 and PLA2R1*,* revealed in IMN, which means that the canonical NF-κB pathway plays an active role in IMN [37]; however, there was no significant difference in the expression of NFKB1 and IRF4 in this study. The shortage may be that the samples from these two groups are too small. Second, focusing on validated target genes may exclude potential targets that have not been validated by the experiment. Third, the antibodies used in this study may have a certain impact on the experimental results, since the results from immunohistochemistry would be better to use antibodies that recognize the phosphorylated forms of PI3K and AKT and not the total form. Fourth, this study explored the relationship between VEGFA and PLA2R-associated IMN, but did not explore the relationship between VEGFA and secondary membranous nephropathy. It is difficult to show that VEGFA is specifically expressed in IMN, which needs more clinical samples to verify. Last, no knock-down or overexpression of core gene was used to confirm the changes.

## Conclusion

The present study suggests that VEGFA might be the origin of the process leading to IMN, and its inhibition could be a possible therapeutic approach. VEGFA is overexpressed in IMN and is an important therapeutic target for IMN, which may lead to vascular hyperpermeability via the PI3K-Akt signalling pathway, resulting in increased filtration of inflammatory factors, complements, and cytokines, eventually contributing to immune complex (IC) and MAC formation in IMN. Moreover, SNARE interactions in vesicular transport are involved in the development of IMN, and further basic studies are needed to validate our results and to illuminate the molecular mechanism in IMN. These results may provide new targets for the treatment of IMN.

## Supplementary Information

**Additional file 1: Supplement Figure 1.** Biological process enrichment analysis of module genes. **Supplement Figure 2.** Cellular component enrichment analysis of module genes. **Supplement Figure 3.** Molecular function enrichment analysis of module genes. **Supplement Figure 4.** KEGG enrichment analysis of modules genes. **Supplement Figure 5.** Expression profile of core genes involved in angiogenesis in GSE115857. The red nodes represent up-regulated DEGs with a *p* value of <0.05 and logFC >1.0; the green nodes represent down-regulated DEGs with a *p* value of <0.05 and logFC <–1.0. **Supplement Table 1.** Profiles and data of patients. **Supplement Table 2.** The top 15 biological process of DEGs. **Supplement Table 3.** The top 15 Kyoto Encyclopedia of Genes and Genomes (KEGG) pathways of DEGs. **Supplement Table 4.** The most significant biological process enriched for the genes involved in six modules. **Supplement Table 5.** Kyoto Encyclopedia of Genes and Genomes (KEGG) pathways enriched for genes involved in 6 modules. **Supplement Table 6.** The top 30 hub genes rank in cytoHubba. **Supplement Table 7.** Gene sets enriched in phenotype high.

## Data Availability

The datasets analysed during the current study are available in the Gene Expression Omnibus (https://www.ncbi.nlm.nih.gov/geo/, GSE 115,857) repository.

## Abbreviations

PLA2R: The M-type phospholipase A2 receptor
IMN: Idiopathic membranous nephropathy
VEGFA: Vascular endothelial growth factor A
VEGFR2: Vascular endothelial growth factor receptor 2
GEO: Gene Expression Omnibus
DEGs: Differentially expressed genes
DAVID: Database for annotation, Visualization and Integrated Discovery
STRING: Search Tool for the Retrieval of Interacting Genes
GSEA: Gene set enrichment analysis
GO: Gene Ontology
BP: Biological process
CC: Cellular component
MF: Molecular function
KEGG: Kyoto Encyclopedia of Genes and Genomes
MCODE: Molecular Complex Detection
SNARE: Soluble N-ethylmaleimide–sensitive factor attachment protein receptor
ECM: Extracellular matrix
SSU-rRNA: Small subunit ribosomal RNA
PI3K: Phosphoinositide 3-kinase
NES: Normalized enrichment score
FDR: False discovery rate
PPI: Protein–protein interaction
CAMs: Cell adhesion molecules
AMPK: Adenosine 5 ‘-monophosphate (AMP)-activated protein kinase
KDIGO: Kidney Disease | Improving Global Outcomes
NRP1: Neuropilin 1
IC: Immune complex
MAC: Membrane attack complex
NFKB1: Nuclear factor kappa B 1
IRF4: Interferon regulatory factor 4
