# Supplementary material for: VEGFA promotes the occurrence of PLA2R-associated idiopathic membranous nephropathy by angiogenesis via the PI3K/AKT signalling pathway
Source: BMC Nephrol. 2022 Sep 16;23:313. doi: 10.1186/s12882-022-02936-y (PMC9482157; doi:10.1186/s12882-022-02936-y)
Supplement: Supplementary file 1 — Additional file 1: Supplement Figure 1. Biological process enrichment analysis of module genes. Supplement Figure 2. Cellular component enrichment analysis of module genes. Supplement Figure 3. Molecular function enrichment analysis of module genes. Supplement Figure 4. KEGG enrichment analysis of modules genes. Supplement Figure 5. Expression profile of core genes involved in angiogenesis in GSE115857. The red nodes represent up-regulated DEGs with a p value of <0.05 and logFC >1.0; the green nodes represent down-regulated DEGs with a p value of <0.05 and logFC <–1.0. Supplement Table 1. Profiles and data of patients. Supplement Table 2. The top 15 biological process of DEGs. Supplement Table 3. The top 15 Kyoto Encyclopedia of Genes and Genomes (KEGG) pathways of DEGs. Supplement Table 4. The most significant biological process enriched for the genes involved in six modules. Supplement Table 5. Kyoto Encyclopedia of Genes and Genomes (KEGG) pathways enriched for genes involved in 6 modules. Supplement Table 6. The top 30 hub genes rank in cytoHubba. Supplement Table 7. Gene sets enriched in phenotype high. [file 12882_2022_2936_MOESM1_ESM.docx]

Supplement Figure 1.


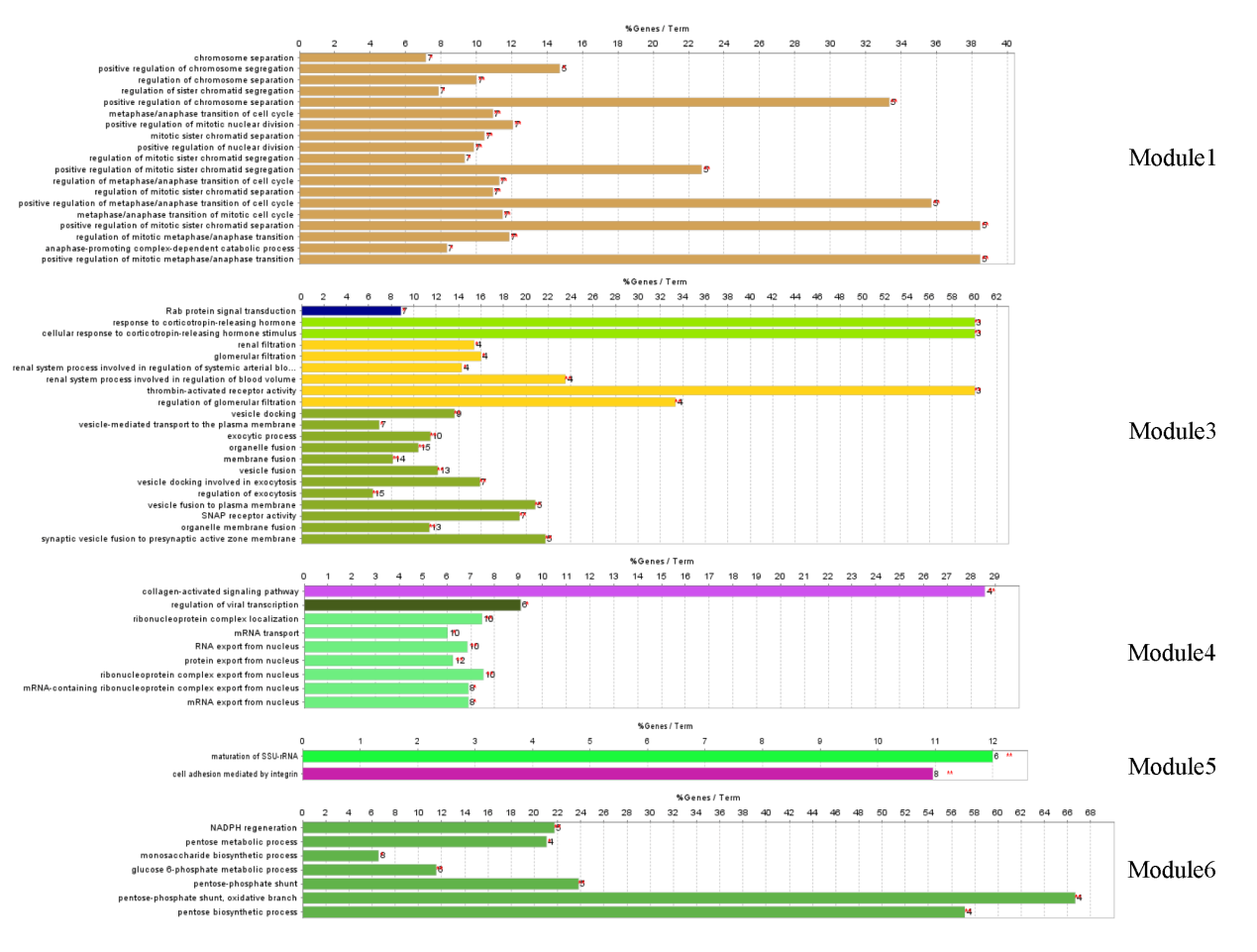


Biological process enrichment analysis of module genes.

Supplement Figure 2.


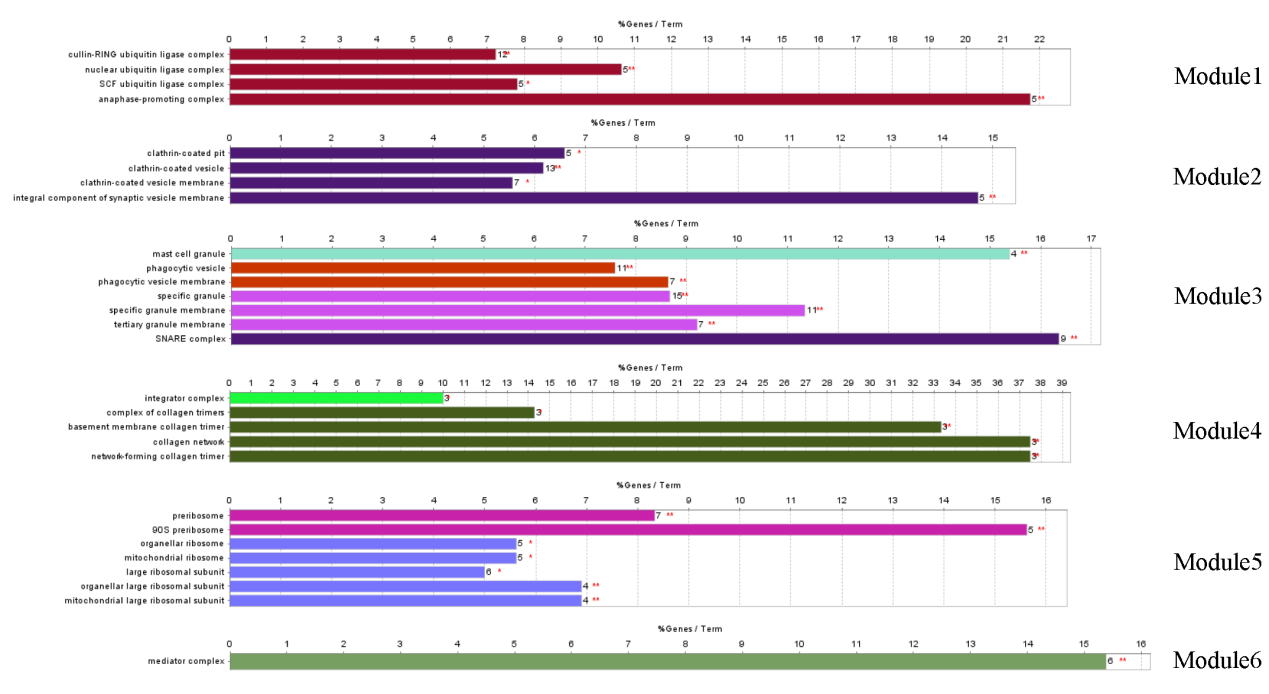


Cellular component enrichment analysis of module genes.

Supplement Figure 3.


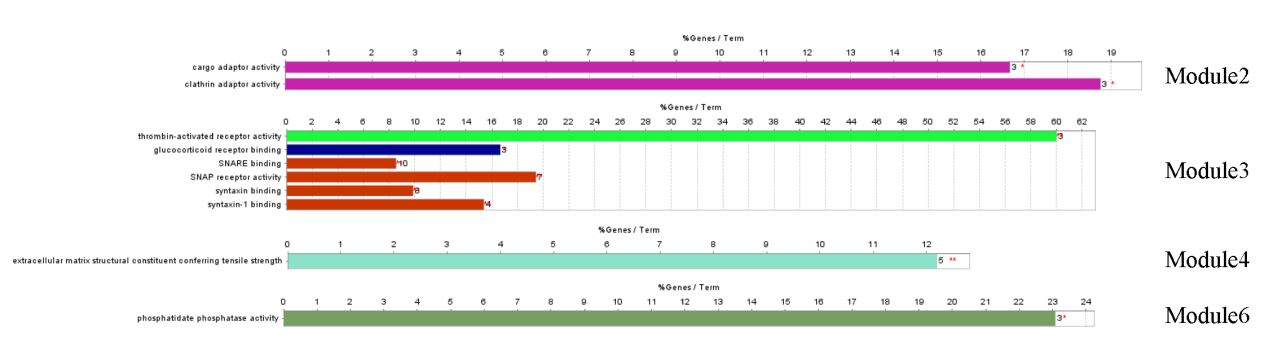


Molecular function enrichment analysis of module genes.

Supplement Figure 4.


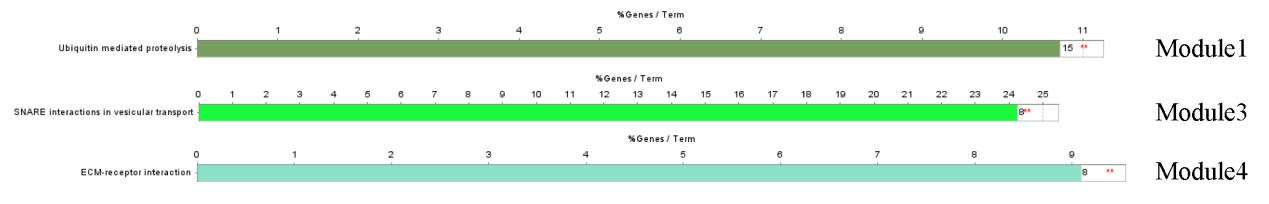


KEGG enrichment analysis of modules genes.

Supplement Figure 5.


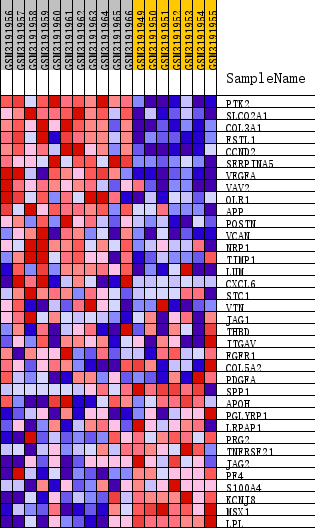


Expression profile of core genes involved in angiogenesis in GSE115857. The red nodes represent up-regulated DEGs with a p value of <0.05 and logFC >1.0; the green nodes represent down-regulated DEGs with a p value of <0.05 and logFC <–1.0.

Table Legends

Supplement Table 1. Profiles and data of patients

| Patient no. | Age | Sex | Anti-PLA2R(RU/mL) | THSD7A(ng/mL) |
| --- | --- | --- | --- | --- |
| 1 | 59 | M | 109.25 | ＜2 |
| 2 | 30 | M | 279.44 | 48.37 |
| 3 | 63 | F | 487.91 | 28.81 |
| 4 | 57 | M | 891.13 | 55.75 |
| 5 | 66 | F | 742.14 | 49.36 |
| 6 | 59 | M | 425.82 | 46.38 |
| 7 | 54 | M | 678.43 | 86.49 |
| 8 | 44 | F | 590.11 | 73.49 |
| 9 | 56 | M | 3.04 | 22.57 |
| 10 | 50 | M | ＜2 | 46.72 |

*When the measured value is <14 Ru /mL: negative; ≥ 14 to < 20 RU/mL: suspected; ≥ 20 Ru /mL: positive.

Supplement Table 2. The top 15 biological process of DEGs

| Description | *P* Value | Count | Gene Symbol |
| --- | --- | --- | --- |
| GO:0006357 regulation of transcription from RNA polymerase II promoter | 4.80E-06 | 54 | FOSL2, MED24, MED23, FOXO3, FOS, ATP2B4, TRAK2, SMARCD3, ZNF444, FOSL1, PITX2, NFKBIZ, SATB2, ARID5A, PKN1, RB1, FOSB, HNF4G, FOXJ3, PURA, RBBP8, SLTM, MED7, VEGFA, SMARCAL1, GLIS3, BBS7, TADA3, ZNF367, SOX7, ABCA2, FOXQ1, LYL1, JUND, CHD1, SUPT4H1, NFATC3, TFDP1, MAFG, SREBF1, KAT2A, MAFF, BRD3, TBX3, MAFB, ZMYM6, CREB1, TFCP2, ECM1, ZBED1, ATF3, RFX1, MAFA, RFX2 |
| GO:0060213 positive regulation of nuclear-transcribed mRNA poly(A) tail shortening | 2.20E-05 | 7 | ZFP36, TNRC6C, BTG2, CPEB3, CNOT1, CNOT7, TNRC6B |
| GO:0007155 cell adhesion | 7.30E-04 | 48 | ITGAL, SPG7, CCL2, SCN1B, NINJ2, ITGB5, CLDN10, POSTN, PCDHGC3, NEO1, CD151, PCDHAC2, SCARF1, VCL, APLP1, VCAM1, CD47, LPXN, WISP2, LAMB3, COL6A6, SORBS2, COL12A1, LAMB1, COL8A1, SPON1, DSCAM, ICAM1, SVEP1, ICAM4, PTPRF, ICAM3, ITGA2, CCL4L1, IGFALS, PCDH17, TPBG, SIRPA, COL4A6, ARVCF, CDH13, PRKD2, SIGLEC5, CD34, CX3CR1, NPHS1, DSC2, CFDP1 |
| GO:1900153 positive regulation of nuclear-transcribed mRNA catabolic process, deadenylation-dependent decay | 0.001235 | 6 | ZFP36, TNRC6C, CPEB3, CNOT1, CNOT7, TNRC6B |
| GO:0034097 response to cytokine | 0.00135 | 11 | FNTB, FOS, IFI27, REL, JUN, JUND, ITIH4, PML, RARA, BCL2L1, FOSL1 |
| GO:0030198 extracellular matrix organization | 0.001421 | 25 | ITGAL, PDGFB, LUM, ADAMTSL4, ITGB5, POSTN, VCAM1, CD47, LAMB3, POMT1, SERPINE1, BCL3, AGRN, LAMB1, COL8A1, VWA1, COL4A4, ICAM1, ICAM4, ICAM3, OLFML2A, ITGA2, NID1, COL4A6, EGFLAM |
| GO:0007165 signal transduction | 0.001834 | 99 | IL9R, TNFSF15, TNFSF14, IL15, CNOT7, CD2AP, ADORA1, UNC5B, CHRNA9, LILRA2, MIER1, PPP1R1B, PDE4B, RARA, CCL4L1, GEM, IL21, TANK, VEGFC, ARRB1, CD34, CSNK1G3, EXT2, ITGAL, CCL2, SP110, MDK, GPR27, TEK, AGRN, NPHP1, MAP2K3, SMAD5, MRC2, SPHK1, NR4A2, NR4A1, ECM1, TRADD, ZNF217, PLCG1, PDE7A, IL5RA, GRB7, PLAU, PDIA3, HINT1, RASSF9, KCNK10, ARHGAP12, LNPEP, WISP2, MKLN1, NR2F6, SHC1, CASP1, LTB, IFNGR1, IRAK1, AVP, ARHGAP28, IGFALS, PKN1, ARRDC3, PRKCD, OR51G2, DAPK1, CD83, TNFRSF10B, TNFRSF10D, ZDHHC13, RIN2, MAP3K10, GNB5, NRGN, RASD1, SRGAP2, THOC1, PRKCZ, GULP1, NR3C2, TRIM13, SCARF1, LPXN, IL10RB, PDE1A, PLA2R1, SECTM1, CAP2, CREB1, ANXA1, MAPK11, DPYSL2, RALGDS, TEX2, SYDE1, IGFBP1, IGFBP2, ARAP2 |
| GO:0044344 cellular response to fibroblast growth factor stimulus | 0.002221 | 8 | ZFP36, HYAL1, EGR3, CCL2, NR4A1, POSTN, KDM5B, GCLM |
| GO:0016477 cell migration | 0.002833 | 22 | PRKCZ, ARC, FGFR4, FMNL3, CCDC88A, PTPRF, CD151, MDK, SDC2, PDPK1, SDC1, PLCG1, HOXA5, SORBS2, GSK3B, ARPC5L, SH3KBP1, TGFBR3, TNN, CSK, USP33, SPATA13 |
| GO:0043123 positive regulation of I-kappaB kinase/NF-kappaB signaling | 0.002881 | 21 | IRAK1, SECTM1, F2RL1, TRIM13, PARK2, PIM2, ECM1, TRADD, TRAF3IP2, CASP10, CTH, TNFRSF10B, REL, MIER1, ZDHHC13, TICAM2, TGM2, PELI2, CASP1, IL1A, F2R |
| GO:0042493 response to drug | 0.003154 | 33 | XPO1, SORD, ABCA2, PPOX, GCLM, MDK, HADHA, B2M, FOS, GAD2, JUND, PEMT, DHODH, CCNO, DNMT3B, FOSL1, SREBF1, ICAM1, SLC8A1, CREB1, ANXA1, ITGA2, DPYSL2, FOSB, CPT1A, SS18, VEGFC, JUN, ABCC4, ABCC1, IGFBP2, HTR2A, ABCC6 |
| GO:0014070 response to organic cyclic compound | 0.003223 | 10 | KAT2A, FNTB, ICAM1, CD83, G6PD, BTG2, LUM, ABCC4, CPT1A, POLR2A |
| GO:0009612 response to mechanical stimulus | 0.003595 | 11 | CCL2, PTGER4, BTG2, JUN, JUND, POSTN, FOSB, IGFBP2, MBD2, PSPH, FOSL1 |
| GO:0050927 positive regulation of positive chemotaxis | 0.003653 | 5 | CDH13, VEGFA, F2RL1, ITGA2, F7 |
| GO:0008285 negative regulation of cell proliferation | 0.003913 | 40 | RARRES1, PML, SOX4, SOX7, MXI1, CNOT7, ADORA1, KANK2, LIF, FNTB, CCL3L1, CCL3L3, PEMT, RARA, AXIN2, CSK, FOSL1, IL1A, CTBP1, DAB2IP, NACC2, PDS5B, CGRRF1, JARID2, VHL, KLF10, RB1, PIM2, FRZB, SLIT3, CDH13, TNFRSF9, VEGFC, BTG2, JUN, SCIN, GDF11, RBM38, MDM4, F2R |

Supplement Table 3. The top 15 Kyoto Encyclopedia of Genes and Genomes (KEGG) pathways of DEGs

| Description | *P* Value | Count | Gene Symbol |
| --- | --- | --- | --- |
| hsa05323 Rheumatoid arthritis | 0.001819 | 15 | TCIRG1, ICAM1, ITGAL, CCL3, CCL2, IL15, ATP6V1C1, FOS, CCL3L1, JUN, TEK, VEGFA, CCL3L3, LTB, IL1A |
| hsa02010 ABC transporters | 0.002041 | 10 | ABCB8, TAP1, ABCC4, ABCC1, ABCA2, ABCB6, ABCA6, ABCG1, ABCC5, ABCC6 |
| hsa04668 TNF signaling pathway | 0.004494 | 16 | ICAM1, CCL2, SOCS3, MAP2K3, CREB1, CREB5, MAPK11, IL15, TRADD, VCAM1, RPS6KA5, LIF, CASP10, FOS, JUN, BCL3 |
| hsa04130 SNARE interactions in vesicular transport | 0.006102 | 8 | STX6, STX3, BET1, SEC22B, VAMP4, VAMP3, VAMP1, STX1B |
| hsa05166 HTLV-I infection | 0.009722 | 28 | ITGAL, XPO1, PDGFB, PPP3R2, ANAPC11, IL15, BCL2L1, VCAM1, FOS, POLE3, NFATC3, FOSL1, TBPL1, NFATC1, ZFP36, KAT2A, ICAM1, EGR2, ANAPC5, CREB1, ANAPC4, CDC23, RB1, CDC27, ATF3, CCND2, JUN, GSK3B |
| hsa04120 Ubiquitin mediated proteolysis | 0.018961 | 17 | ANAPC5, SOCS3, VHL, ANAPC4, PML, CDC23, KEAP1, ANAPC11, PARK2, UBE2Q2, CDC27, PRPF19, WWP2, UBA3, SIAH1, PIAS2, CUL4B |
| hsa05132 Salmonella infection | 0.020675 | 12 | FOS, CCL3, CCL3L1, JUN, ARPC5L, CCL3L3, PKN1, CCL4L1, MAPK11, CASP1, IL1A, IFNGR1 |
| hsa04514 Cell adhesion molecules (CAMs) | 0.025698 | 17 | ICAM1, ITGAL, PTPRF, VTCN1, ICAM3, CD276, NTNG1, NTNG2, CLDN10, NEO1, CLDN11, SDC2, CLDN15, VCAM1, SDC1, CD34, ICOS |
| hsa04380 Osteoclast differentiation | 0.026566 | 16 | FOSL2, SOCS3, CREB1, PPP3R2, MAPK11, FOSB, SIRPA, FOS, LILRA2, JUN, JUND, FOSL1, IFNGR1, IL1A, SYK, NFATC1 |
| hsa04064 NF-kappa B signaling pathway | 0.028321 | 12 | VCAM1, ICAM1, IRAK1, PLCG1, TICAM2, CCL4L1, TNFSF14, BCL2L1, LTB, PLAU, TRADD, SYK |
| hsa04151 PI3K-Akt signaling pathway | 0.050168 | 32 | FGFR4, PDGFB, ITGB5, BCL2L1, FOXO3, PDPK1, LAMB3, COL6A6, TEK, GYS1, TNN, LAMB1, PPP2R2B, SYK, PPP2R1B, COL4A4, SGK1, CREB1, NR4A1, ITGA2, PKN1, CREB5, COL4A6, VEGFC, CCND2, GSK3B, VEGFA, GNB5, EFNA5, THEM4, PPP2R3C, F2R |
| hsa04512 ECM-receptor interaction | 0.061638 | 11 | COL4A4, CD47, LAMB3, SDC1, COL6A6, ITGB5, ITGA2, TNN, AGRN, LAMB1, COL4A6 |
| hsa04152 AMPK signaling pathway | 0.063824 | 14 | PPP2R1B, SREBF1, CPT1C, SCD, CREB1, ACACA, CREB5, FOXO3, CPT1A, PDPK1, GYS1, PPP2R2B, RAB10, PPP2R3C |
| hsa05031 Amphetamine addiction | 0.06951 | 9 | FOS, ARC, PPP1R1B, JUN, CAMK2G, CREB1, PPP3R2, CREB5, FOSB |
| hsa05142 Chagas disease (American trypanosomiasis) | 0.083357 | 12 | PPP2R1B, IRAK1, FOS, CCL3, CCL2, CCL3L1, JUN, CCL3L3, SERPINE1, MAPK11, PPP2R2B, IFNGR1 |

Supplement Table 4. The most significant biological process enriched for the genes involved in six modules

| Description | *P* Value | Count | Gene Symbol | Module |
| --- | --- | --- | --- | --- |
| GO:0045842 positive regulation of mitotic metaphase/anaphase transition | 0.00 | 5 | ANAPC11, ANAPC4, ANAPC5, CDC23, CDC27 | Module 1 |
| GO:0048284 organelle fusion | 0.00 | 15 | ANXA1, BET1, CPLX2, PRKN, RAB20, RAB8B, SEC22B, STX1B, STX3, STX6, SYT9, TFRC, VAMP1, VAMP3, VAMP4 | Module 3 |
| GO:0003093 regulation of glomerular filtration | 0.00 | 4 | ADORA1, F2R, F2RL1, PDGFB | Module 3 |
| GO:0071426 ribonucleoprotein complex export from nucleus | 0.00 | 10 | AGFG1, MAGOHB, NUP153, NUP155, RAE1, RIOK2, SRRM1, THOC1, WDR33, XPO1 | Module 4 |
| GO:0033627 cell adhesion mediated by integrin | 0.00 | 8 | ICAM1, ITGA2, ITGB5, LPXN, PLAU, PLPP3, SERPINE1, SYK | Module 5 |
| GO:0030490 maturation small subunit ribosomal RNA (SSU-rRNA) | 0.00 | 6 | HEATR1, KRI1, NOL11, RIOK2, SRFBP1, WDR3 | Module 5 |
| GO:0009051 pentose-phosphate shunt, oxidative branch | 0.00 | 4 | G6PD, PGD, PHGDH, UBL4A | Module 6 |

Supplement Table 5. Kyoto Encyclopedia of Genes and Genomes (KEGG) pathways enriched for genes involved in 6 modules

| Description | *P* Value | Count | Gene Symbol | Module |
| --- | --- | --- | --- | --- |
| KEGG:04120 ubiquitin mediated proteolysis | 0.00 | 15 | ANAPC11, ANAPC4, ANAPC5, CDC23, CDC27, CUL4B, KEAP1, PML, PRKN, PRPF19, SIAH1, SOCS3, UBA3, UBE2Q2, VHL | Module 1 |
| KEGG:04130 soluble NSF Attachment Protein Receptor (SNARE) interactions in vesicular transport | 0.00 | 8 | BET1, SEC22B, STX1B, STX3, STX6, VAMP1, VAMP3, VAMP4 | Module 3 |
| KEGG:04512 extracellular matrix (ECM)-receptor interaction | 0.00 | 8 | CD47, COL4A4, COL4A6, COL6A6, ITGA2, ITGB5, LAMB1, SDC1 | Module 4 |

Supplement Table 6. The top 30 hub genes rank in cytoHubba

| Betweenness | Bottleneck | Eccentricity | EPC | MNC |
| --- | --- | --- | --- | --- |
| ALB | ALB | NACC2 | ALB | ALB |
| VEGFA | VEGFA | JUN | JUN | JUN |
| JUN | EPRS | POLR2A | VEGFA | VEGFA |
| EPRS | FOS | ANAPC4 | FOS | FOS |
| POLR2A | JUN | RPL10 | SOCS3 | POLR2A |
| ALDH18A1 | ALDH18A1 | CDC27 | CCL2 | HIST2H2BE |
| GSK3B | GSK3B | FOS | ICAM1 | CREB1 |
| CREB1 | CREB1 | RPSA | CREB1 | CCL2 |
| FOS | RB1 | PLCG1 | KEAP1 | ICAM1 |
| RAB11A | VCL | RB1 | POLR2A | EPRS |
| TFRC | BCL2L1 | CASP2 | VCAM1 | SOCS3 |
| BCL2L1 | RAB11A | AXIN2 | PARK2 | POLR2L |
| VCL | LMNA | G6PD | HIST2H2BE | TFRC |
| RB1 | CD34 | PPP2R1B | VHL | B2M |
| HIST2H2BE | SLC35A2 | HIST2H2BE | BCL2L1 | RAB11A |
| B2M | POSTN | VEGFA | GSK3B | KEAP1 |
| RPSA | SREBF1 | CCND2 | B2M | BCL2L1 |
| SOCS3 | GAD2 | CD34 | CDC27 | HIST1H2BN |
| G6PD | G6PD | SMARCD3 | SHC1 | VCAM1 |
| KEAP1 | IRAK1 | XPO1 | SIAH1 | RPSA |
| ARRB1 | RTN4 | MAPK11 | CDC23 | CDC27 |
| CTTN | CCL2 | DPYSL2 | TFRC | GSK3B |
| CCL2 | CYFIP1 | RTN4 | FBXL19 | SHC1 |
| EIF4G1 | SPHK1 | NR4A2 | ANAPC11 | ARRB1 |
| SREBF1 | KEAP1 | KDM1A | CD34 | PARK2 |
| CD34 | PSMA4 | HIST1H2BN | FBXL5 | VHL |
| XPO1 | CTTN | CASP1 | KCTD6 | RPLP0 |
| RPLP0 | CCND2 | EPRS | ASB6 | CTTN |
| KDM1A | SUCLG2 | EFNA5 | ANAPC4 | FBXL19 |
| SYK | POU5F1 | PML | FBXW2 | AVP |

Supplement Table 7. Gene sets enriched in phenotype high

| Name | ES | NES | NOM p-val | FDR q-val |
| --- | --- | --- | --- | --- |
| HALLMARK_ANGIOGENESIS | 0.4953875 | 1.7208529 | 0.012320329 | 0.1586698 |

FDR: false discovery rate; NES: normalized enrichment score; NOM: nominal. Gene sets with NOM P-val <0.05 and FDR q-val <0.25 are considered as significant.
